# Late Presentation of HIV Infection in the Netherlands: Reasons for Late Diagnoses and Impact on Vocational Functioning

**DOI:** 10.1007/s10461-018-2082-9

**Published:** 2018-03-17

**Authors:** S. E. M. van Opstal, J. S. van der Zwan, M. N. Wagener, S. K. Been, H. S. Miedema, P. D. D. M. Roelofs, E. C. M. van Gorp

**Affiliations:** 1 Center of Expertise Innovations in Care, Rotterdam University of Applied Sciences, Rochussenstraat 198, 3015 EK Rotterdam, The Netherlands; 2 Erasmus MC, Department of Internal Medicine and Infectious Diseases, University Medical Center Rotterdam, Rotterdam, The Netherlands; 3 Erasmus MC, Department of Viroscience, University Medical Center Rotterdam, Rotterdam, The Netherlands

**Keywords:** HIV, Late diagnosis, Late-presenters, Vocational functioning

## Abstract

Late diagnosis of HIV remains a major challenge in the HIV epidemic. In Europe, about 50% of all people living with HIV are diagnosed late after infection has occurred. Insight into the reasons for late diagnoses is necessary to increase the number of early diagnoses and optimize treatment options. This qualitative study explored the experiences of 34 late-presenters through in-depth semi-structured interviews. A variety of reasons for late diagnoses emerged from our data and led to a division into four groups, characterized by two dimensions. Regarding vocational functioning, the consequences of late diagnoses were health-related problems prior to and since diagnosis, and problems concealing the HIV status. Healthcare providers should offer HIV tests to groups at risk, and be alert for clinical HIV indicator conditions. It is recommended to increase awareness of HIV transmission routes, symptoms and tests, and the benefits of early testing and early entry to HIV care.

## Resumen

Diagnóstica tarde de los pacientes con el virus de l’immunodéficienca humana (VIH) siguen siendo un importante desafío en la lucha contra la epidemia del VIH. En Europa, sobre 50% de todas las personas que viven con VIH se diagnostican tarde después de la infección ha ocurrido. Se necesita entender las razones de la diagnóstica tarde, para optimalizar el número de pacientes diagnosticados temprano del VIH y optimalizar el tratamiento de pacientes con VIH. Este estudio cualitativo explora las experiencias de 34 pacientes diagnosticados tarde de VIH por medio de entrevistas semiestructuradas profundas. Varias razones para diagnóstica tarde han surgido de nuestra base de datos. Estas razones condujeron a una separación en cuatro grupos que a su vez pueden ser caracterizado por dos dimensiones. En cuanto a las consecuencias de la infección de VIH para la función en el trabajo algunos principales problemas de salud fueron descubiertos. Estés problemas se presentaron antes y durante el proceso de diagnóstica y por lomas problemas relacionados con el estado del VIH. Proveedores de atención médica tendrían que ofrecer pruebas del VIH a grupos de riesgo y prestar atención a indicadores clínicos del VIH. Se recomienda crear conciencia de las rutas posibles de transmisión de VIH, los síntomas, las capacidades de prueba, los beneficios del diagnóstico precoz del VIH y entrada temprana al tratamiento de VIH.

## Introduction

Since the introduction of combination antiretroviral therapy, an increasing number of people living with HIV (PLWH) are able to achieve viral suppression. As a result, the mortality rates of PLWH have dropped by 50% [1]. However, despite medical advances in both treatment and access to care, early testing for HIV infection remains a challenge [2]. In Europe, about 50% of all PLWH are diagnosed late after infection has occurred [3]. The most common definition of a late diagnosis is based on a CD4 cell count < 350 cells/mm^3^ at the moment of presentation at a healthcare facility, or presenting with an AIDS-defining event [4]. Late diagnosis of HIV is a major risk for both individuals and society. It is associated with increased morbidity and a mortality rate that is tenfold during the first year after diagnosis, when compared to non-late presenters [5, 6]. In addition, long-term consequences of late diagnosis include an increased risk of neurocognitive impairment and persistent incomplete CD4 cell count restoration [7, 8]. Moreover, unawareness of the HIV infection before diagnosis, leads to an increased risk of onward transmission and is, therefore, a key factor in the spread of HIV [9]. Finally, late diagnoses have an economic impact, due to higher resource use and more clinical events [6, 10, 11].

In the Netherlands, 52% of the PLWH are diagnosed late after infection; a relatively high proportion compared with other European countries [3, 12]. Several studies examined risk factors for a late diagnosis in the Netherlands and found that heterosexual transmission, older age, and South-East Asia/sub-Saharan Africa as regions of origin, are associated with a late diagnosis [13, 14]. However, these risk factors do not indicate the actual barriers for early testing and the underlying causes of late diagnoses. Therefore, insight into the reasons for a late diagnosis is needed to increase the number of early diagnoses and optimize treatment options.

Besides lack of clarity about the reasons for late diagnoses, HIV professionals in the Netherlands have reported another important issue: the consequences of late diagnoses of HIV on daily functioning and, especially, on vocational functioning. Since HIV affects many people of working age, problems in vocational functioning have become a major concern. Maintaining employment and returning to work are challenging for PLWH [15]. The unemployment rate of PLWH is significantly higher, compared to the general population [16, 17]. Furthermore, HIV could lead to absenteeism, loss of productivity and skills, and discrimination at work [16–19]. Particularly due to the high morbidity risk after diagnosis, late-presenters might face specific employment-related concerns. Thus, to improve supportive care for PLWH in the Netherlands, a research project (the TREVI study) was started to explore the experiences of professionals and PLWH with employment-related concerns [18, 20, 21]. To increase insight into the employment-related concerns among PLWH, the question arose to explore the experiences of late-presenters in this area as part of the TREVI study. We therefore conducted a qualitative study with the following questions: (1) “What are the reasons for late diagnoses from the perspective of late-presenters in the Netherlands?” and (2) “Which consequences of HIV are experienced by late-presenters in their vocational functioning?” These insights are important to be able to reduce the number of late diagnoses and improve counselling after diagnosis.

## Methods

### Study Design

This qualitative study was conducted in one of the main HIV treatment centers of the Netherlands (Erasmus Medical Center; EMC). Our research design was based on the grounded theory approach, with a cyclical process of data collection and analyses to explore the perspectives and experiences of late-presenters [22]. The study complied with The Netherlands Code of Conduct for Scientific Practice, from the Association of Universities in the Netherlands [23]. Our study was exempted from a medical ethical review, in accordance with the Dutch Medical Research Involving Human Subjects Act [24].

### Participant Selection

A purposive sampling strategy was used to select respondents. Late-presenters were eligible when they met the definition of late presenter which is based on the CD4 cell count < 350 cells/mm^3^ at the moment of presentation at a healthcare facility, or presenting with an AIDS-defining event. Moreover, late-presenters were considered for qualification if they were aged ≥ 18 years, able to speak Dutch or English, diagnosed with HIV infection < 3 years ago, and in care at the EMC. All eligible persons who had an appointment for a consultation during the period of data collection were invited to participate. They were invited before or after the consultation at the HIV treatment center by their HIV physician, face-to-face or by telephone. Persons willing to participate were contacted, informed about the objectives of the study, and asked for their written consent to participate.

### Data Collection

Experiences of the participants were explored through in-depth semi-structured interviews. The topic list was based on current insights from scientific literature on late diagnosis of HIV, and working with HIV (Table 1). All interviews were conducted in Dutch or English, with a maximum of two researchers present at each interview. Author SO conducted the interviews, together with eight research assistants from the Rotterdam University of Applied Sciences, who were under the supervision of SO, who was present at the first two interviews of each assistant. Data collectors conducted no more than one interview each day and took notes during and after each interview. The interviews were conducted face-to-face at the HIV treatment center or by telephone, depending on the preference of the participant. Interviews were recorded on audiotape and transcribed *ad verbatim*. Although it is impossible to ascertain the full extent to which rapport was established during data collection, the openness of the participants and the richness of the collected data on this sensitive topic, suggest that the participants felt comfortable during the interviews. Participants were compensated for travel expenses, if such costs had been incurred.

**Table 1:** Topic list interviews

**Reasons for late HIV diagnoses**
Risk perception
Symptoms prior to diagnosis
Fear for diagnosis and/or consequences
Knowledge about HIV prior to diagnosis
Contact with medical professionals prior to diagnosis
**Consequences of HIV for vocational functioning**
Impact in social situations
Impact in vocational functioning
*Additional topics (second cycle of data collection)*
Employment status prior to and after diagnosis
Problems at work due to HIV
Absenteeism prior to or after diagnosis
Adaptations at work
**Suggestions to prevent late diagnoses**
Suggestions for professionals
Suggestions for (undiagnosed) patients

Data were collected in two cycles. The first cycle was from November 2011 to January 2012. After exploring the first 27 interviews, it was concluded that additional data were needed on the consequences of a late diagnosis of HIV for vocational functioning. Four topics were added to the topic list for this purpose (Table 1). The second cycle of data collection was from November 2013 to January 2014. Data were collected until data saturation occurred.

### Data Analysis

The data analysis was based on a cyclical process [25]. After the first cycle of data collection the first three authors individually read the transcripts of the interviews and analyzed the interviews in an exploratory way. Based on that analysis, the topic list was adjusted to create more depth on certain topics. After the second cycle of data collection, our data was thoroughly analyzed by encoding in three steps; i.e. open, axial and selective encoding. During the process, analyses were discussed with the other co-authors. We used the qualitative software program ATLAS-Ti 7 for encoding. The first step was performed individually, whereas the authors collaborated for the following two steps. Coded texts and emerging relationships between codes were discussed among the authors. Individual coding was discussed to reach consensus about the codes. Texts were re-coded when necessary. Subsequently, all codes were rearranged into categories related to background characteristics, reasons for late diagnoses, consequences for vocational functioning, and suggestions to prevent late diagnoses. A summary of the results was made by author SO and discussed among the authors to reach consensus on the findings.

For the description of the background characteristics of the respondents, region of origin was defined by the country of birth. Educational attainment indicated the highest level of education that was completed and was divided into three categories: low (no education, primary or lower secondary education, and lower vocational education), middle (secondary and middle vocational education) and high (higher vocational education, university or higher).

### Member Check

A member check was performed in December 2016 through January 2017 to confirm the results and the conclusions drawn from them. We asked four recently diagnosed (< 3 years ago) late-presenters for the member check, in which we discussed a summary of our findings to confirm that our collected data and findings were accurate, complete and up-to-date.

### Research Team

All authors have a background in healthcare and are experienced in either patient care of PLWH, or research in PLWH. Authors MW, PR, HM and EG were involved in the development of a multidisciplinary, evidence-based guideline on HIV and work, which also included qualitative studies [18, 20].

The first author (SO) is a female PhD student at a HIV treatment center not involved in patient care. The first three authors were involved in all steps of the data analysis, and work as researchers (SO and MW) or as research assistant (JZ). They were not involved in patient care and have a range of experience in qualitative and quantitative research. The research assistants were students of various healthcare studies and underwent training on qualitative research methods and fundamentals of HIV/AIDS. Author (SB) helped in selecting/approaching respondents for the interviews. All authors were involved in discussions about the interpretation of the analyzed data.

## Results

### Characteristics of the Participants

A total of 51 eligible HIV-infected late-presenters were invited to participate in the study. Of them, 17 refused to participate, due to one of the following reasons: too busy (n = 8), fear for participating in a research project (n = 2), emotional burden to talk about HIV (n = 2), or an unknown reason (n = 5). Thus, 34 respondents were included and interviewed either face-to-face (n = 31) or by telephone (n = 3). The interviews lasted between 20 and 80 min, with an average duration of 42 min. The mean age of the respondents was 46.6 years (range 23–75 years). Most respondents were male (n = 30) and more than half (n = 19) were homosexual and of Dutch origin (Table 2).

**Table 2:** Characteristics of the study population

Characteristic	n = 34
*Gender*	
Male	30
*Sexual orientation*	
Homosexual	19
Heterosexual	11
Bisexual	4
*Transmission route*	
Men having sex with men	23
Heterosexual	9
Blood or blood products	1
Unknown	1
*Educational attainment*	
High	13
Middle	11
Low	9
Unknown	1
*Employment status*	
Paid employment	20
Volunteer work	1
Retired	3
Unemployed	8
Unknown	2
*Region of origin*	
The Netherlands	19
Western Europe (other than NL)	2
Latin America	5
The Caribbean	2
Sub-Saharan Africa	3
Other	3
*CD4 cell count at the moment of HIV diagnoses*	
0–200 cells/mm^3^	25
200–350 cells/mm^3^	9

### Reasons for Late Diagnoses

A variety of reasons for late diagnoses were described by respondents, ranging from ‘feeling healthy’ to ‘symptoms that were not linked to HIV by a professional’ and ‘fear for the consequences of a HIV diagnosis’. Generally, a combination of reasons led to the late diagnosis. Patterns in combinations of reasons for a late diagnosis emerged from our data and led to a division into four groups of late-presenters, distinguished by two dimensions. The first dimension is based on awareness or unawareness of the fact that the respondent has been at risk of a HIV infection. The second dimension is based on the presence or absence of HIV-related symptoms, prior to the diagnosis.

All respondents could be assigned to one of the four groups, according to a combination of these two dimensions. Although the combination of reasons for each group of late-presenters is unique, similarities occurred between groups sharing one of the dimensions (Table 3). For example, ‘Feeling healthy’ as a reason is shared by both groups with the dimension ‘No symptoms prior to diagnosis’. Out of 34 respondents, 19 had symptoms prior to their diagnoses and 22 respondents were aware of the fact that he/she had been at risk of a possible HIV infection. The four groups of late-presenters and the combination of reasons of each group are described below.

**Table 3:** Late-presenters and their combination of reasons for late diagnosis

	No symptoms prior to diagnosis	Symptoms experienced prior to diagnosis
**Unaware of the fact that they have been at risk of HIV infection**	*There was no reason to be tested (n = 7)* – In a stable relationship and therefore unaware of a possible risk of HIV transmission through their partner – Lack of knowledge about the possible transmission routes of HIV – Feeling healthy	*People saw no reason to be tested, despite symptoms (n = 5)* – In a stable relationship and therefore unaware of a possible risk of HIV transmission through their partner – Lack of knowledge about the possible transmission routes of HIV – The symptoms were not experienced as severe – The symptoms were not linked to HIV by the respondent – The symptoms were not recognized by professionals as HIV-related – A previous negative test
**Aware of the fact that they have been at risk of HIV infection**	*People were aware of a reason to be tested, but no or little need was experienced (n = 8)* – Perception of low risk behavior for HIV – Feeling healthy – Fear for the possible consequences of the diagnosis – Being unfamiliar with the Dutch healthcare system	*People were aware of a reason to be tested, but did not take any action (n = 14)* – Perception of low risk behavior for HIV – The symptoms were not experienced as severe – The symptoms were not linked to HIV by the respondent – The symptoms were not recognized by professionals as HIV-related – A previous negative test – Fear for the possible consequences of the diagnosis – Being unfamiliar with the Dutch healthcare system

#### Unaware of the Risk and no Symptoms experienced Prior to Diagnosis

7 respondents were unaware of the fact that they had been at risk of a possible HIV infection, and experienced no symptoms prior to their diagnosis. For this group, there was no direct reason to be tested. The most important reasons for late testing in this group are described below.

##### Stable Relationship

All respondents in this group were in a stable relationship. The main route of HIV transmission was through partners who had had sexual contact with others. One respondent stated: “I got infected with HIV through my partner—now my ex-partner. We were together for three years and we had unsafe sex because I trusted him.”

##### Lack of Knowledge About HIV Transmission Routes

Another reason to be unaware of the risk was lack of knowledge about possible HIV transmission routes. This was due to very limited sexual education in the country of origin or, in one case, mental disability. One respondent described his unawareness as follows: “My knowledge about HIV was very little, I didn’t know that it could be the consequence of the pleasure (referring to sexual contact)”. Another respondent answered the question about how she was infected with HIV: “I don’t know, maybe when I was swimming 1 day—I was bitten by a fish and became really sick”. The lack of knowledge led to unawareness and was therefore a reason for late diagnoses.

##### Feeling Healthy

Many respondents described ‘feeling healthy’ as an important reason for their late diagnosis: “I had no symptoms at all, so why would I go to see a doctor?”. Another respondent stated: “A hospital is for people who are sick… I felt okay so I didn’t have to go there”.

#### Unaware of the Risk, but Symptoms experienced Prior to Diagnosis

5 of the 34 respondents experienced symptoms prior to the HIV diagnosis, but were unaware of the fact that they had been at risk of HIV infection. Reasons for a late diagnosis were partly similar to the previously described group; having a stable relationship or lack of knowledge about HIV transmission routes were also important barriers in this group. Additional reasons are described below.

##### Symptoms Were not Experienced as Severe

The symptoms that were present prior to diagnosis were often experienced as rather mild and vague, e.g. occasionally a skin rash, fatigue, or a cold. If the symptoms were mild and did not interfere with their daily functioning, respondents saw no reason to be tested: “Everybody is sick once in a while and I’ve always been able to do my job, so I didn’t have any reason to see my general practitioner”.

##### Symptoms not Linked to HIV by the Respondents

The symptoms that were present were not directly related to HIV by the respondents. Instead, symptoms were often attributed to comorbidities, aging, a stressful event, or other causes: “I had some problems with my health before the diagnosis, but I thought that was the result of chemotherapy”, and “I’ve had a lot of ear inflammations, but I’m a frequent diver”. Some symptoms were already familiar to the respondents before the HIV infection: “I had recurrent skin rashes, but I used to have eczema as a child, so I didn’t pay much attention to it”, and “I was often really tired, but I’ve always been someone who needs to sleep a lot”.

##### Symptoms not Recognized by Healthcare Professionals as HIV Related

When healthcare professionals were involved, symptoms were often not recognized as HIV-related symptoms. One respondent described: “I had pneumonia several times and shingles, but they only diagnosed COPD”. Another respondent stated: “My skin was full of rashes, but I was taking medication for depression and one of the side-effects was rashes. So, everyone, including the doctors, thought it was just a side-effect”. Respondents described that some professionals only considered the symptoms of their own specialty: “I had a type of eczema that was common for HIV-positive patients, I was sweating at night and I had several infections on my throat. The dermatologist and ear, nose and throat physician knew about all the symptoms, but no one suggested further examination”.

Having a previous negative HIV test was mentioned once in this group as a reason for a late HIV diagnosis. Because this was frequently mentioned in the group in which the respondents were aware and experienced symptoms prior to diagnosis, the reason ‘A previous negative HIV test’ is described in that group.

#### Aware of the Risk, but no Symptoms Experienced Prior to Diagnosis

8 of the 34 respondents experienced no symptoms prior to diagnosis, but were aware that they had been at risk of HIV infection. Respondents in this group often refer to themselves as being ‘’naïve or foolish” to have ignored the risk. They were aware of the reason to be tested but did not feel the necessity to be tested, mostly because they did not have any symptoms. Therefore, feeling healthy is a reason for late testing, similar to the first described group. A respondent stated: “I was healthy, so I thought everything was alright. That sounds like a stupid reason, but that’s the reason why I didn’t go to my general practitioner”.

##### Perception of Low-Risk Behavior for HIV

Most of the respondents experienced their own risk of HIV infection as low. They did not consider themselves as part of a risk group for HIV, or thought that their behavior had not been too risky: “I thought I was smart enough. You know—you should limit the number of sexual partners and be careful with needles … but I’ve never used any drugs and I’m not promiscuous. So, I thought I would be okay”. Some of the respondents had one, or only a few occasions for possible HIV transmission. For example, a married man stated: “I only cheated on her once and my wife is not infected, so it must be from this one night with another woman”.

##### Being Unfamiliar with the Dutch Healthcare System

A few respondents stated that unfamiliarity with the healthcare system was a reason for their late diagnosis. They were ignorant about the possibilities to be tested, did not know where to go, or were afraid of possible high costs. In addition, one respondent stated that acquiring a residence permit had more priority than his health, even though he was motivated to be tested: When I arrived as a refugee, I knew I needed a test because of the abuse. I tried to explain, but it was so difficult. There were some tests when I arrived in the Netherlands, but I don’t know the results or if it was an actual HIV-test […] First I had to find a place to live, then I would look for a general practitioner”.

Fear for the consequences of HIV diagnosis was mentioned a few times in this group, but will be described in the next group since, for these latter respondents, it was one of the main reasons for late diagnosis.

#### Aware of the Risk and Symptoms Experienced Prior to Diagnosis

Slightly less than half of the respondents (n = 14) were aware that they had been at risk of HIV infection and had experienced symptoms prior to their diagnosis. Respondents in this group were mostly aware of the necessity of an HIV-test; nevertheless, they reported several reasons for postponing testing (Table 3). Some consciously ignored the symptoms they experienced and the fact that they had been at risk, because they feared the consequences of the diagnosis. Their ignorance was supported by the fact that many of the symptoms they experienced were not linked to HIV by the professionals involved, or were not severe for a prolonged period. Some of the respondents in this group felt that they had a low risk of possible HIV infection, and others stated that they already anticipated a HIV infection, but were consciously postponing a test.

##### Fear for the Possible Consequences of the Diagnosis

The respondents in this group often mentioned that fear for what could happen after the diagnosis was an important reason for their late diagnosis. Many respondents feared several possible consequences of the diagnosis, e.g. side-effects of medication and financial consequences: “I was afraid of the costs involved, because my health insurance would probably not cover it”. Another respondent stated: “A reason to postpone the test was that I had heard that HIV medication causes a lot of side-effects”. Fear for the social impact of HIV-related discrimination was frequently mentioned as a reason for postponing a test: “I was really afraid of negative reactions. What are people going to say and do? Are they going to exclude me? I really didn’t want to have this disease”. One respondent was afraid that the diagnosis would reveal his cheating which would have an impact on his entire family: “There are so many consequences. I’ve been cheating on my wife and I have a family. So, if you can postpone it…”.

##### A Previous Negative HIV Test

A previous negative HIV test was mentioned a few times by respondents as a reason for their late diagnosis. Respondents described that they experienced the risk of HIV infection after the test as low, especially if they were in a stable relationship or had had only one sexual partner since the test.

### Consequences of Late Diagnosis for Vocational Functioning

Several consequences of the HIV infection for vocational functioning were described by the respondents. Below, we describe the consequences related to a late diagnosis.

#### Health Problems Prior to Diagnosis Influencing Vocational Functioning

More than half of the respondents (n = 19) experienced symptoms prior to their diagnosis. They were mostly unaware of the fact that their symptoms were presumably caused by the HIV infection. Some had experienced symptoms for years prior to the diagnosis. Frequently mentioned health problems prior to the diagnosis were fatigue, general malaise, oral infections, pneumonia, and dermatological problems. These symptoms influenced productivity at work and led to increased absenteeism: ‘’Once I had a long-lasting inflammation of my throat. I was used to getting better in 3 days but suddenly noticed that it lasted longer than a week. That was annoying—especially for my work’’. Another respondent described: “The year prior to the diagnosis I was frequently sick. I had fever a few times and had to call in sick quite often. When I was able to work, I felt tired and was unable to do as much as I used to do”.

#### Health Problems Since Diagnosis Influencing Vocational Functioning

Many respondents described their health as poor when they were diagnosed. For 25 of the 34 respondents, their CD4 cell count was ≤ 200 cells/mm^3^ at the time of diagnosis which, for some respondents, meant that they were not allowed to work: “My CD4 cell count was lower than 200, it was only 100—so I wasn’t allowed to work. Actually, I haven’t worked for 5 weeks now”.

Respondents regularly described that their health was so poor due to opportunistic infections, they had to stay in the hospital for a few weeks or months to recover after the diagnosis: “I was in the hospital for 2 months because of very severe pneumonia, with 2 weeks intensive care. Now it’s been almost 5 months since the diagnosis, but I’m still too weak to return to work”. A few respondents stated they were afraid of losing their job because they had to call in sick for a longer period and did not want to disclose their HIV status.

Some respondents stated they were shocked by the low CD4 cell count and then had psychological problems influencing their work: “When I heard my CD4 cell count, I started to plan my own funeral. I was so afraid of dying. I tried to work as a way of distraction, but wasn’t able to concentrate at all”. While the recovery was described as slow by many respondents, most described an improvement in their health status after diagnosis: “I’m less tired, full of energy now. I’ve actually never felt so good in years”.

#### Problems Concealing Their HIV Status

Some respondents described they had problems concealing their HIV status because their physical health was poor at the time of diagnosis: “Because I was so sick at the moment of diagnosis, I had to stay in the hospital for 8 weeks. I told my employer I had pneumonia, but I realize this is hard to believe. I wish I had got tested before, then I could have prevented this”.

### Suggestions to Prevent a Late Diagnosis

#### Providing Information

Many respondents stated that providing more information about HIV is necessary for the prevention of late diagnoses. More knowledge about the possible transmission routes and the fact that, nowadays, the HIV virus can be successfully suppressed by medication is important information that could stimulate early testing. One respondent described: “It’s important that people know that there is medication for HIV. You don’t have to die because of the HIV, but you do have to go to the doctor […] For me, I didn’t know, I thought I was going to die”. Furthermore, it is important to provide information about where people can be tested and the costs involved.

#### Alertness of Healthcare Professionals

Many respondents described that they had symptoms prior to their diagnosis that are known for their association with HIV, and had visited their general practitioner (GP) with these symptoms. Some of them visited specialists with these symptoms, e.g. a dentist, dermatologist, or ear, nose and throat physician. Respondents stated that GPs should be more alert for these symptoms, especially when they are recurrent, or when patients belong to a risk group for HIV infection. Many respondents described that their GP should have been more proactive in offering a HIV test, even if the respondents and the GP thought that the risk for HIV infection was low.

#### Stimulate People to Take Their Own Responsibility

Many respondents emphasized that people should assume their own responsibility and realize the importance of regular testing. The media should also stimulate/report that people should be tested on a regular basis, even when they are in a stable relationship or think that the risk for HIV infection is low. Respondents also stated that HIV testing should be facilitated in smaller cities nationwide, with the possibility of anonymity and with little or no costs involved.

### Results of Member Check

A summary of the results was discussed with four recently diagnosed late-presenters; all could identify themselves with the results. No new themes emerged, but some themes were further elaborated.

## Discussion

This study explored reasons for late testing from the perspective of late-presenters in the Netherlands and the consequences they experienced in their vocational functioning. A variety of reasons for late diagnoses emerged. From the perspective of the late-presenters, mostly a combination of reasons led to their late diagnoses. Four main patterns of reasons were discovered among individual late-presenters, distinguished by two dimensions: whether or not the respondent was aware of the fact that he/she had been at risk of a HIV infection, and whether the respondent experienced symptoms prior to the diagnosis. Although the combination of reasons for each group was unique, similarities occurred based on a shared dimension. The consequences described by the late-presenters for their vocational functioning were mainly associated with poor health status prior to and shortly after diagnosis. Because they were diagnosed late, many of the respondents had been struggling with their health for months or years, which resulted in absenteeism and decreased productivity at work. At the moment of diagnosis, the majority of respondents experienced severe symptoms due to opportunistic infections caused by decreased immune status. Many respondents were unable to work in the period after their diagnosis because they were hospitalized or needed to convalesce. In addition, it was more difficult to conceal the HIV status at work due to the presence of symptoms.

The group of late-presenters who were unaware that they had been at risk for HIV infection and did not experience any symptoms prior to their diagnosis, is the most challenging group in the prevention of a late diagnosis. Respondents of this group did not appear to have a reason to be tested. All were in a stable relationship, which is a recognized risk factor for a late diagnosis [26], i.e. an HIV test felt unnecessary for these respondents. It is noteworthy that half of this group were women and the majority had a heterosexual transmission route; this transmission route was reported by others to be a risk factor for late diagnosis [13, 14]. In accordance with previous studies, in our respondents being asymptomatic was also identified as reason for late diagnosis [26–28]. Although the respondents experienced no symptoms and had no clinical indicator conditions for HIV, all respondents of this group belonged to a high-risk population for HIV, i.e. they were either from a HIV endemic area, or were men who had sex with men. It has been suggested that HIV testing could be cost-effective if the undiagnosed HIV prevalence is ≥ 0.1% [29–31]. In our respondents coming from a HIV endemic area, in all their countries of origin the HIV prevalence was ≥ 1%, which suggests that proactively offering HIV tests in this group is likely to be cost-effective [32]. Therefore, for new migrants, asylum seekers and refugees from countries with a HIV prevalence > 1%, routine screening for HIV is recommended. Centers such as the Dutch Health Center for Asylum Seekers offer these tests already regardless of residence status or health insurance. However, it is recommended to implement these as routine tests and to inform potential HIV patients about the existence of these possibilities.

The vast majority of respondents who had symptoms prior to their diagnosis, but were unaware of the fact that they had been at risk for a HIV infection, had visited their GP with these symptoms. Research shows that it is common that PLWH visit their GP in the year prior to their diagnoses, indicating that opportunities to diagnose HIV have been missed [33–35]. Studies also show that healthcare providers experience various barriers to offer a HIV test, e.g. anxiety to address the issue of an HIV test, regarding the diagnosis HIV as uncommon, or expecting the disease only within known risk groups for HIV [10, 36]. In addition, symptoms of HIV tend to be unspecific (e.g. fatigue and weight loss) or could be related to comorbidities, which is a known risk factor for late diagnosis of HIV [28, 33]. About half of the respondents described visiting their GP prior to their diagnosis with symptoms that are known as clinical indicator conditions for HIV testing, with recurrent pneumonia, herpes zoster and sexually-transmitted diseases most frequently described. Furthermore, the vast majority of respondents had additional indications for recommending an HIV test such as, men having sex with men, or originating from an HIV endemic area, which are indicators for offering an HIV test according to national and European guidelines [37, 38]. To reduce the number of late-presenters, GPs should be stimulated, in their guidelines, to offer HIV tests proactively and be more alert for HIV indicator conditions, particularly in risk groups for HIV. Following the diagnosis of HIV indicator conditions in line with the European guideline is important, as this approach has led to significant decreases in the probability of late HIV diagnosis [39]. Special attention is required for migrant groups, as a lack of knowledge about possible transmission routes emerged from our data, especially in people who were of non-Dutch origin. This lack of knowledge led to unawareness of a risk for HIV infection and indicates a need for sexual education and information about HIV, particularly for migrants.

Late-presenters who were aware of the risk for HIV infection, but experienced no symptoms prior to their diagnosis, mostly felt it was unnecessary to be tested. In accordance with similar studies, perceiving oneself to be at low risk of HIV was also identified in our study as an important barrier for testing [26, 27, 40–44]. Late-presenters in this group should be stimulated in targeted municipal health education programs to assume responsibility for an HIV test; moreover, it is important to raise awareness of the risk for themselves and for others, as well as the benefits of early HIV testing and early entry to HIV care. Furthermore, respondents’ lack of knowledge on the symptoms of HIV and the efficiency and possible side-effects of HIV medication were identified as a reason for not testing in our study and previous studies, indicating a clear need for effective information to recognize these symptoms [26, 45].

Structural barriers, such as a lack of knowledge about locations for HIV testing, experiencing problems in communication with healthcare professionals, and worries about medical insurance and possible costs of testing, were mentioned a few times; this is in accordance with other studies [28, 36, 40, 43, 46, 47]. The present study only included persons who were able to speak Dutch and English. Special attention is needed for persons experiencing structural barriers in healthcare, especially those unable to speak/read Dutch or English. Therefore, it is necessary to offer counseling in this area and to guide asylum seekers into the healthcare system.

The largest group of respondents in our study experienced symptoms prior to their diagnosis and were aware of the fact that they had been at risk for a HIV infection. Similar to the previous group who were aware, but experienced no symptoms, respondents in this group should be motivated in targeted (online) public health campaigns to assume responsibility for an HIV test and become aware of the risks of postponing HIV testing for themselves and others. Fear for the consequences was an important barrier for an HIV test in this group, as found in earlier studies [28, 36, 40, 42, 43, 46, 48]. Especially the social consequences and stigma were frequently mentioned as a reason for their late diagnosis. Besides motivating persons to test for HIV who are aware of a possible HIV infection, it is important that healthcare professionals and policymakers make a strong commitment to stop/prevent stigmatization.

One of the strengths of our study is the variety of patients regarding region of origin, transmission route and educational attainment, which resulted in a sample comparable with the total Dutch HIV population [49]. However, in other countries the group of PLWH infected through blood products or infected needles is larger compared with that in the Netherlands [12, 50–53]. These transmission routes lead to unawareness of a possible HIV infection and are therefore an important risk factor for late diagnoses, as also confirmed by others [13]. Offering HIV tests to patients who are injecting drug users or who have received blood products in the past in HIV endemic areas, could help to avoid late diagnoses.

Another limitation is that all our respondents were receiving care from Erasmus MC and living in/close to one of the largest cities in the Netherlands. This could make access to healthcare facilities easier, because the density of Community Health Services, and the possibility of anonymous HIV testing without consulting a GP, is higher in Rotterdam than in rural areas. Therefore, additional barriers to accessing health care could have been missed, e.g. that HIV testing is time consuming, or problems with transportation.

### Implications of Late Diagnosis for Vocational Functioning

Our findings point out that optimal functioning at work is especially problematic when PLWH are coping with physical symptoms, as also reported by others [15, 54]. Due to the association between late diagnoses and a high risk of morbidity, late-presenters are at increased risk for vocational problems. These problems aggravate the existing economic and social impact of late diagnoses of HIV. Stigma plays a larger role for late-presenters due to the problems with concealing their HIV status caused by the presence of opportunistic infections. The problems in vocational functioning for late-presenters lead us to the conclusion that counseling in this area should be provided, taking the special needs of late-presenters into account. Because of their central position in the Dutch healthcare system, it is recommended that GP’s as well as HIV nurses have to play a central role in this area [20].

## Conclusion

The importance of an early diagnosis of HIV is well established. A variety of reasons for late diagnoses from the perspective of late-presenters emerged from our data. Several different approaches are needed to address the causes of and prevent late diagnoses in the future. When patients are unaware of the risk for HIV infection, healthcare providers have to play a central role by offering tests in risk groups for HIV and being alert for clinical HIV indicator conditions. Especially, when it comes to general practitioners. Patients who are aware of the risk for HIV infection should be stimulated to assume their responsibility. Targeted public health campaigns should be provided to raise awareness of HIV transmission routes, the risks of late diagnoses, and the benefits of early HIV testing and entry to HIV care. Information on HIV symptoms and medication, about test locations, costs involved and test procedures, should be easily available and accessible. Social stigma remains an area requiring attention. After diagnosis, vocational counseling should be offered, specifically addressing the needs of late-presenters.
