# Correction to: Late Presentation of HIV Infection in the Netherlands: Reasons for Late Diagnoses and Impact on Vocational Functioning

**DOI:** 10.1007/s10461-018-2253-8

**Published:** 2018-08-24

**Authors:** S. E. M. van Opstal, J. S. van der Zwan, M. N. Wagener, S. K. Been, H. S. Miedema, P. D. D. M. Roelofs, E. C. M. van Gorp

**Affiliations:** 1 Center of Expertise Innovations in Care, Rotterdam University of Applied Sciences, Rochussenstraat 198, 3015 EK Rotterdam, The Netherlands; 2 Erasmus MC, Department of Internal Medicine and Infectious Diseases, University Medical Center Rotterdam, Rotterdam, The Netherlands; 3 Erasmus MC, Department of Viroscience, University Medical Center Rotterdam, Rotterdam, The Netherlands

## Correction to: AIDS and Behavior (2018) 22:2593–2603 10.1007/s10461-018-2082-9

The original version of this article was published open access. Unfortunately, due to a technical issue, the copyright holder name in the online version (HTML and XML) is incorrectly published as “Springer Science + Business Media, LLC, part of Springer Nature 2018”. Instead, it should be “The Author(s) 2018”.

The original article has been corrected.

